# FDup: a framework for general-purpose and efficient entity deduplication of record collections

**DOI:** 10.7717/peerj-cs.1058

**Published:** 2022-09-06

**Authors:** Michele De Bonis, Paolo Manghi, Claudio Atzori

**Affiliations:** Istituto di Scienza e Tecnologie dell’Informazione “A. Faedo” (ISTI), Consiglio Nazionale delle Ricerche (CNR), Pisa, Italy

**Keywords:** Deduplication, Scholarly communication

## Abstract

Deduplication is a technique aiming at identifying and resolving duplicate metadata records in a collection. This article describes FDup (Flat Collections Deduper), a general-purpose software framework supporting a complete deduplication workflow to manage big data record collections: metadata record data model definition, identification of candidate duplicates, identification of duplicates. FDup brings two main innovations: first, it delivers a full deduplication framework in a single easy-to-use software package based on Apache Spark Hadoop framework, where developers can customize the optimal and parallel workflow steps of blocking, sliding windows, and similarity matching function via an intuitive configuration file; second, it introduces a novel approach to improve performance, beyond the known techniques of “blocking” and “sliding window”, by introducing a smart similarity matching function T-match. T-match is engineered as a decision tree that drives the comparisons of the fields of two records as branches of predicates and allows for successful or unsuccessful early-exit strategies. The efficacy of the approach is proved by experiments performed over big data collections of metadata records in the OpenAIRE Research Graph, a known open access knowledge base in Scholarly communication.

## Background

Deduplication is a technique for the identification and purging of duplicate metadata records in a collection of records, generally intended as sets of field-value pairs describing the properties of an entity. To this aim, deduplication techniques first identify groups of equivalent records in a collection (*i.e.*, different records describing the same entity), then apply preferred duplication resolution strategies (*e.g.*, record merge, record purging, *etc*.) to return a disambiguated collection of records. The deduplication process entails several known challenges, which have to do with (Atzori, Manghi & Bardi, 2018): (i) the efficiency of the process, which in the case of “big data” metadata record collections is severely biased by the quadratic complexity derived from the need of matching every record with all the records in the collection; (ii) the similarity matching function and its ability to encode and capture record equivalence; and (iii) the complexity and flexibility of tools, often resulting from assembling different pieces of code, not easily adaptable to deal with different scenarios.

The efficiency challenge is traditionally tackled by adopting a combination of two heuristics: clustering and sliding window  (Paulo & Pereira, 2014; Venish & Sankar, 2015). Clustering makes sure potentially equivalent records are grouped into clusters (aka “blocks”), within which the pair-wise similarity match will be quadratically applied. Blocking reduces the number of matches but, most importantly, allows for the parallel execution of the process across different blocks (Manghi et al., 2020). The sliding window technique further optimizes the number of matches within the individual blocks by sorting the records in such a way that similar records are likely kept close to each other and then matching each record with the “k” following records (“K-length window”).

The similarity matching challenge is typically addressed with a function yielding an equivalence score between two records, obtained as the weighted sum of the scores calculated by the pair-wise comparison of records’ field values. The flexibility of the function depends on the available comparators, predicates, *etc*., the configuration of the weights for each comparator/field, *etc*.

As mentioned above, customization and configuration of such tools can be cumbersome and a barrier for developers, whose steep learning curve typically leads to the re-implementation of new tools or “one-shot” code to patch/assemble existing projects.

This article presents FDup (Flat Collections Deduper), a software framework based on the Apache Spark Framework that supports the full deduplication process as described above. FDup improves the current state-of-the-art approaches in two ways:

• *Customization and flexibility of configuration*: users can flexibly and easily customize the various phases of the framework via a single configuration file: clustering, sliding window and similarity functions; it offers a set of predefined comparators which can be used to configure a deduplication process without being proficient in programming languages;
• *Efficient Similarity Matching*: users can configure a similarity function T-match, defined as a *PublicationTreeMatch* which drives the comparisons of the fields of the records and allows to configure early-exit strategies to further reduce the overall performance.

FDup was conceived as an enhancement of the GDup framework (Graph Deduper) (Atzori, Manghi & Bardi, 2018), a software framework designed to deduplicate big data graphs, intended as sets of record collections connected by relationships. GDup adopts parallel clustering and sliding windows techniques to identify duplicates and delivers strategies to resolve duplication while preserving the topology of the graph. In order to further improve performance, GDup was endowed with the T-match function, somehow pioneering performance optimization in the similarity matching phase. The resulting software modules, efficiently identifying duplicates in a flat collection of records, have been factored out of the GDup software package and published as FDup, a stand-alone software package (De Bonis, Atzori & La Bruzzo, 2022) that can be easily re-used in such common scenarios.

FDup is today in use in the production system of the OpenAIRE infrastructure to deduplicate the entities of the OpenAIRE Research Graph (http://graph.openaire.eu) (Manghi et al., 2021). The OpenAIRE Research Graph is a scholarly communication “big data graph” whose goal is to support the realization of research impact monitoring and discovery services for funders, institutions, and researchers in specific disciplines. The graph is constructed by harvesting, harmonizing, and deduplicating beyond 300Mi+ records about publications, datasets, software, and organizations harvested from 97,000 scholarly communication sources (*e.g.*, journals, institutional repositories, data repositories). The simplicity of use and the improved performance derived from FDup will be demonstrated by experimenting with two collections of 10Mi and 230Mi of non-deduplicated publication records in the graph.

*Outline*: the last part of this section analyses the state-of-the-art to highlight the innovation gap brought by FDup. The ‘Material’ section formally presents the functional architecture of FDup, focusing on the requirements, the model adopted, and the technical implementation of the framework, providing an example of its usage in the OpenAIRE infrastructure. ‘Methods’ describes methods and techniques used to test both the efficiency and the general-purposeness of the framework defining an innovative custom configuration for the deduplication. ‘Results’ provides experimental results and highlights how FDup overcomes traditional approaches in terms of time consumption. ‘Discussion’ and ‘Conclusions’ conclude the article and delve into possible future works and developments of the framework.

### State of the art

Many tools and frameworks contributed in different ways to the general problem of entity linking of which deduplication is a specific application. Complete surveys of such approaches can be found in Nentwig et al. (2017) and Sitas & Kapidakis (2008). This class of problems has been deeply studied in the literature, and many solutions were proposed to specifically tackle the usability and the efficiency of the approaches. Solutions focus mainly on the optimization of the quadratic complexity by accurately selecting (*e.g.*, heuristics) the pairs of entities to be matched and by parallelizing the actual match operations Rahm & Peukert (2019). Interestingly, the survey also analyzes the approaches used for similarity matching of a pair of records. Record matching is in general driven by similarity measures that are computed atomically but never mentioned as a phase where further optimization of the overall process can take place.

Saltzer & Hylton (2002) propose a clustering algorithm that tolerates errors and catalogues variations by using a search engine and an approximate string matching algorithm. The approach proved to be effective as it identifies more than 90 percent of the related records and includes incorrect records in less than 1 percent of the clusters.

Tauer et al. (2019) describe a solution based on a graph partitioning formulation that improves the accuracy of entity resolution by incrementally revising results whenever new information about the input entities is provided. The approach improves accuracy and optimizes the process by reducing the number of comparisons required in subsequent rounds of deduplication.

The GDup framework described in Atzori, Manghi & Bardi (2018), offers an out-of-the-box solution to a complete workflow of deduplication for big data knowledge graphs. The framework includes ground truth management, candidate identification *via* blocking and sliding windows, identification and merging of duplicates, and graph topology consolidation. The Spark-based implementation drives the parallelization process, further boosting the performance introduced by clustering. The similarity match function can be flexibly configured but does not support any optimization option.

A novel contribution to performance optimization is offered by Papadakis et al. (2019), where block purging and filtering techniques are adopted to further reduce the number of records in a block, hence the number of matches. These techniques take advantage of the frequency to which a pair of entities appear in the same group to avoid redundant and rare comparisons. The approach shows better performance than traditional blocking/sliding window techniques, but it is not recommended when a high recall is required.

In most approaches, the similarity function is provided as a weighted mean of the sum of comparators applied to the pair of records without performing any optimization process. For example, in Azeroual et al. (2022), the authors present a six-step deduplication process in which the comparison between two entities is driven by a similarity vector. Such vector is represented as an aggregation of single similarity scores between attributes and it is subsequently used to apply rules defining threshold-based conditions for the equivalence of the entities. Similarly, in the context of data association, the research in Brown & Hagen (2003) proposed a solution to link criminal records that possibly refer to the same suspect. This method is based on the calculation of a total similarity measure as a weighted sum of the similarity measures of all corresponding feature values. Moreover, Wang, Chen & Atabakhsh (2004) propose a record linkage algorithm for detecting deceptive identities by combining personal attributes scores into an overall similarity score. To draw a conclusion, it establishes a threshold for match decisions using a set of identity pairs labeled by an expert.

In summary, existing approaches tackle performance optimization by optimizing the deduplication workflow phases that precede the similarity match, in some cases renouncing precision due to low recall. FDup follows the same approach by providing an easy-to-use Apache Spark-based framework for deduplication but further improves performance by introducing a similarity function T-match, capable of further reducing execution time without renouncing recall.

## Material

This section describes the FDup architecture in ‘Architecture’ and its software implementation in ‘Implementation’. The former illustrates the deduplication workflow steps and the underlying concepts and features. The latter presents the software modules implementing the architecture steps, referring to the packages published in Zenodo.org by  De Bonis, Atzori & La Bruzzo (2022).

### Architecture

**Figure 1:**
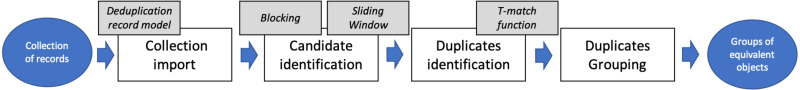
FDup deduplication workflow.

FDup realizes the deduplication workflow shown in Fig. 1. The workflow is intended to deduplicate very large collections of records, processing them in four sequential phases: *Collection import*, to set the record collection ready to process; *Candidate identification*, to cluster the records to be matched; *Duplicates identification*, to efficiently identify pairs of equivalent records via the T-match function, and *Duplicates grouping*, to identify groups of equivalent records via transitive closure.

#### Collection import

FDup operates over a set of flat records, whose structure consists of a set of labels (custom name) and values (extracted by the original record). Therefore, the first step of the workflow consists in mapping the target collection of records, which may not be flat, onto an FDup flat collection of records with labels [*l*_1_, …, *l*_*k*_], which will be used as the template for the configuration of the deduplication steps.

In this article, the experiments are conducted over a collection of bibliographic records for scientific publications as provided by the OpenAIRE Research Graph, featuring the following flat structure:

• *PIDs*: a sequence of values denoting persistent identifiers of the records, *i.e.*, unique identifiers; each record may have more than one PID, released by different agencies at the moment of depositing the article, such as DOIs in Crossref, ArXiv identifiers, PubMed identifiers, *etc*.;
• *title*: the title of the article;
• *abstract*: the abstract of the article;
• *authors*: the list of authors and contributors of the article, provided as a list of strings typicaly following different formats, *e.g.*, “J. Smith”, “Smith, J.”, “Smith, John H.”, “J.H. Smith”;
• *date*: the date of publication of the article, harmonized to a common format dd/mm/yyyy;
• *venue*: the venue of publication, such as the conference or a journal, typically comes as a string of free text.

#### Candidate identification

The ideal similarity matching process, where every pair of records is confronted to yield in an equivalence score, features quadratic complexity and known performance issues. Two common solutions are the techniques of clustering/blocking and sliding window. Both methods apply heuristics to identify, prior to record matching, a selection of record pairs in the collection that are candidates for equivalence.

Clustering functions are of the form *clusteringKey* ([*l*_1_, …, *l*_*k*_]) and are applied to all records in the initial collection to produce one or more keys per record. Functions should be smart enough to ensure that potentially equivalent records likely return the same key. Records are then grouped by key into blocks, and pair-wise comparisons are executed within such blocks. For the scientific publication records above, a reasonable clustering function is one that generates n-grams (fragments of n characters of a string) from the title words or one that generates the PIDs (especially the DOI) when these are available.

Sliding window methods introduce a further optimization of the number of comparisons. Records in one block are sorted by a key (typically obtained from a value of the record’s field) (*orderField*) to obtain an array that is visited from the first element to the last. At every iteration, the pivot record is matched with the subsequent ones in a limited *K* interval (window). The key used for the sorting should be generated in such a way that similar records are likely close in the ordering, possibly within the window range *K*.

FDup offers the possibility to easily configure every single step previously described via single configuration profile, including: a pre-defined and extendable set of *clusteringKey* functions, the *orderField*, and the length *K* of the sliding window.

#### Duplicate identification: T-match function

Duplicate identification consists of a similarity function that matches the equivalence conditions between two records in a block. As such, it is defined by matching the values of the records fields in such a way domain-specific conditions of equivalence are met.

For scientific publication records, OpenAIRE defines two records as equivalent when they describe the same scientific work, hence one object for the purpose of measuring impact. For example, different depositions of the same article in distinct repositories (*e.g.*, ArXiv.org, Zenodo.org) are to be considered equivalent, *i.e.*, cannot be counted as two independent scientific results. For the same reason, two different versions of a document are to be considered different, as they denote different efforts. For example, version 1 and version 2 of a project deliverable.

In real case scenarios, like the one of OpenAIRE, where metadata is harvested from highly heterogeneous data sources, many of the field values in the records are not and cannot be sufficiently harmonized to the degree of uniformity required to make them reliable in the process of equivalence check. This is the case for *abstract*, for which establishing a distance and weight for it is not trivial, and *venue* whose values can hardly be harmonized into comparable values. Moreover, the *date* field cannot be used as a discriminator, as different versions of the article, published at different times, may indeed be regarded as equivalent.

Record equivalence is assessed by relying on three fields, *PID*, *title*, and *authors*, when matching the following considerations:

• **Equivalence by identity**: when two records have one PID in common, they are equivalent;
• **Equivalence by value**: when PIDs are not matching, the records may still be equivalent (*e.g.*, deposited in different repositories), hence a field value equivalence matching is required.

While equivalence by identity is rather straightforward, equivalence by value requires context-driven rules, which in the case of publication match in OpenAIRE are: (i) a rather high *title* equivalence confidence of 99%, as indeed typos may occur; (ii) ensuring that the 0,01% of difference in the titles is not due to a number or Roman number denoting different versions of the publication; and, (iii) making sure the records have corresponding authors, by checking their names.

The majority of deduplication frameworks in the literature encodes record similarity match conditions *via* a similarity function of the form:

\begin{eqnarray*}f([{v}_{1},\ldots ,{v}_{k}],[{v}_{1}^{{^{\prime}}},\ldots ,{v}_{k}^{{^{\prime}}}])=\sum _{i:0\ldots k}{f}_{i}({v}_{i},{v}_{i}^{{^{\prime}}})\times {w}_{i} \end{eqnarray*}

where the *v*_*i*_’s are the values of field *l*_*i*_, ${f}_{i}({v}_{i},{v}_{i}^{{^{\prime}}})$ are *comparators*, functions measuring the “distance” of *v*_*i*_ and ${v}_{i}^{{^{\prime}}}$ for the field *l*_*i*_, and *w*_*i*_’s are the weights assigned to the comparators *f*_*i*_’s, such that ∑_*i*:0…*k*_*w*_*i*_ = 1.

As a result, *f* returns a value in a given range, *e.g.*, [0…1], scoring the distance between two records. The records are regarded as equivalent if the distance measure is greater than a given threshold.

For the example above, the similarity function *PublicationWeightedMatch*, created using the GDup framework in OpenAIRE, encodes both equivalence by identity and by value as follows:

\begin{eqnarray*}\begin{array}{@{}ll@{}} \displaystyle PublicationWeightedMatch(r,{r}^{{^{\prime}}})=&\displaystyle jsonListMatch(r.PIDs,{r}^{{^{\prime}}}.PIDs)\times 0.5\\ \displaystyle &\displaystyle +~TitleVersionMatch(r.title,{r}^{{^{\prime}}}.title)\times 0.1\\ \displaystyle &\displaystyle +~AuthorsMatch(r.authors,{r}^{{^{\prime}}}.authors)\times 0.2\\ \displaystyle &\displaystyle +~LevenshteinTitle(r.title,{r}^{{^{\prime}}}.title)\times 0.2 \end{array} \end{eqnarray*}

where *jsonListMatch*, applied to the field PID, returns 1 if there is at least one PID in common in the two records; *TitleVersionMatch*, applied to the titles, returns 1 if the two titles contain identical numbers or Roman numbers; *LevenshteinTitle* returns 1 if the two (normalized) titles have a Levenshtein distance greater than 90%, and *AuthorsMatch* performs a “smart” matching of two lists of author name strings and returns 1 if they are 90% similar (the minimal equivalence threshold is computed over a manually validated ground truth of equivalent records). All comparators return 0 if their condition is not met. The minimal threshold for two records to be equivalent is 0.5, the threshold that can be reached by *jsonListMatch* alone or by combining the positive results of the three functions *TitleVersionMatch*, *AuthorsMatch*, and *LevenshteinTitle*.

All *f*_*i*_’s in *PublicationWeightedMatch* are computed at the same time and averagely require a constant execution time, despite the successful or unsuccessful match that those may feature. Motivated by such observation, FDup introduces a similarity match function T-match that returns an equivalence match exploiting a decision tree, nesting the comparator functions. Each tree node verifies a condition, which can be the result of combining one or more comparators, and introduces a positive (*MATCH*) or negative (*NO_MATCH*) *exit strategy*. If the exit strategy is not fired, T-match heads to the next node. An early exit skips the full traversal of the tree and can turn the result into a *MATCH*, *i.e.*, a *simRel* relationship between the two records is drawn, or into a *NO_MATCH*, *i.e.*, no similarity relationship is drawn.

A T-match decision is formed by a tree of *named nodes* with outgoing *edges*. The core elements of a T-match node are the *aggregation* function, the list of *comparators*, and a *threshold* value. The aggregation function collects the output of the comparators and delivers an “aggregated” result based on one of the following functions: maximum, minimum, average, and weighted mean. Each comparator in a node accepts two values of the input records for a given field and returns a value in the range 0…1. Notably, different comparators in an aggregation can refer to different fields, giving high degree of customization to end-users (in the following, one node will encode a weighted mean function as the one described above). The execution of a T-match node must end with a decision, which may be:

• *positive*, *i.e.*, the result of the aggregation function is greater or equal than the threshold value;
• *negative*, *i.e.*, the result of the aggregation function is lower to the threshold;
• *undefined*, *i.e.*, one of the comparators cannot be computed (*e.g.*, absence of values); a node also bears a flag *ignoreUndefined* that ignores the *undefined* edge even if one of the values is absent.

For each decision, the node provides the *name* of the next node to be executed. By default, T-match provides two nodes *MATCH* and *NO_MATCH* to be used to force a successful or unsuccessful early exit from the tree.

**Figure 2:**
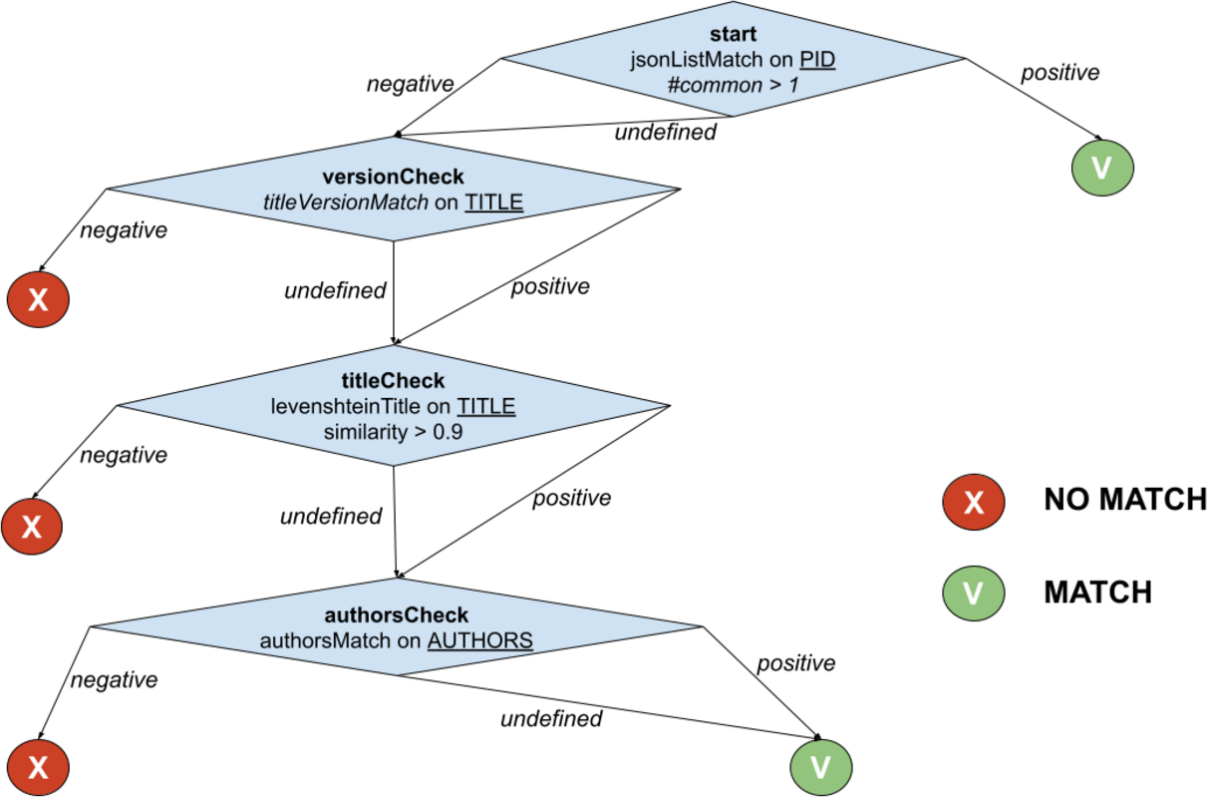
T-match’s decision tree for *PublicationTreeMatch*.

The example in Fig. 2 shows the function *PublicationTreeMatch*, which uses the same comparators but exploiting a T-match decision tree. The individual matches are lined up by introducing *MATCH* conditions early in the process, *i.e.*, equivalence by identity via *PIDMatch*, and then ordering *NO_MATCH* conditions by ascendant execution time, *i.e.*, equivalence by value via *versionMatch*, *titleMatch*, and *authorsMatch*.

Independently of the domain, smart identification of exit strategies becomes a means for developers to reduce the overall deduplication time. Moreover, T-match allows for the definition of multiple paths, hence the simultaneous application of alternative similarity match strategies in one single function. The experiments described in later sections will show that when the number of records is very large, T-match significantly improves the overall performance of the deduplication process.

#### Duplicates grouping

The outcome of the duplicate identification phase is the graph resulting from combining the input collection of records with the set of *simRel* relationships between them. The duplicate grouping phase first finds the sets of equivalent records by calculating the connected components in the graph via the transitive closure of the *simRel* relationships; for example, *A* *simRel* *B* and *B* *simRel* *C* identify the group *A*, *B*, *C*. Secondly, it generates a new graph, where the groups of equivalent records are all linked with a *mergeRel* relationship to a *representative record*, created by the process to identify the groups; for example, the group of nodes *A*, *B*, *C* will deliver the graph of four nodes *A*, *B*, *C*, *R* with the relationships *A* *mergeRel* *R*, *B* *mergeRel* *R*, *C* *mergeRel* *R*.

### Implementation

**Figure 3:**
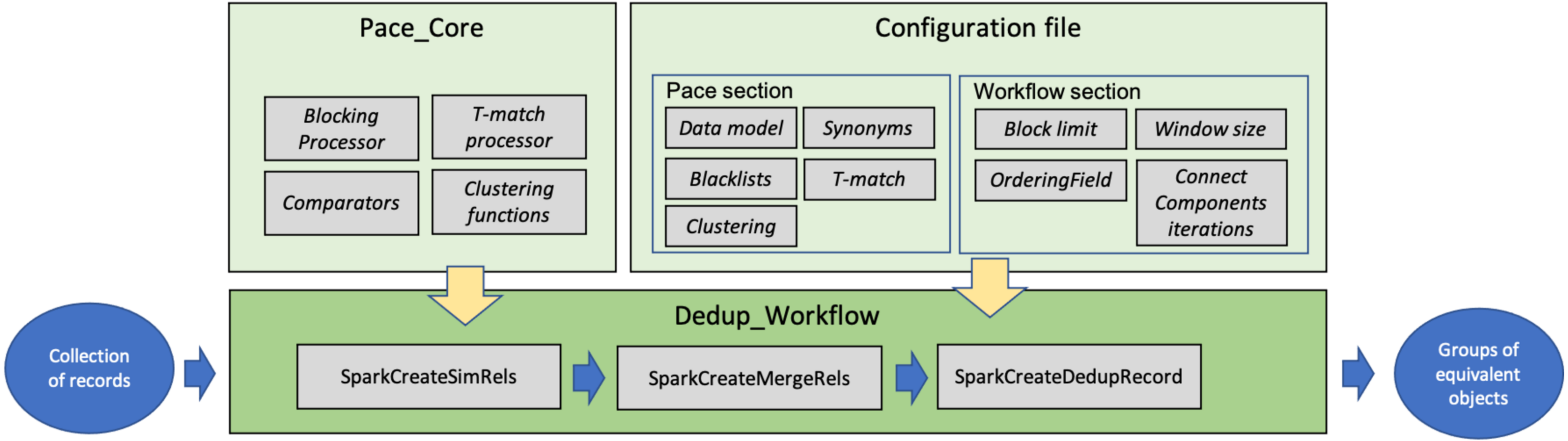
FDup software modules.

This section describes the software modules implementing FDup’s functionalities (GitHub, https://github.com/miconis/fdup) (De Bonis, Atzori & La Bruzzo, 2022) as described in the previous section. FDup’s software is structured in three modules, Pace_Core, Dedup_Workflow, and Configuration file depicted in Fig. 3. The framework is implemented in Java and Scala, and grounds on the Apache Spark Framework, an open-source distributed general-purpose cluster-computing framework. FDup exploits Apache Spark to define record collection parallel processing strategies that distribute the computation workload and reduce the execution time of the entire workflow. Scala is instead required to exploit the out-of-the-box library for the calculation of a “closed mesh” in GraphX (Apache Spark GraphX, https://spark.apache.org/graphx/).

The three modules implement the following aspects of FDup’s architecture:

• Pace_Core includes the functions implementing the candidate identification phase (blocking and sliding window) and the T-match function, as well as the (extensible) libraries of comparators and clustering functions.
• Dedup_Workflow is the code required to build a deduplication workflow in the Apache Spark Framework by assembling the functions in Pace_core according to the comparators, clustering functions, and parameters specified in the Configuration file.
• Configuration file sets the parameters to configure the deduplication workflow steps, including record data model, blocking and clustering conditions, and T-match function strategy.

In the following sections, the three modules are described in detail.

#### The Configuration file

The FDup’s configuration file is expressed in JSON format and consists in two different sections: pace, which defines the T-match function parameters; and workflow, which defines the deduplication workflow parameters.

##### Pace section.

The section specifies the configuration for the pair-wise comparisons, including the *data model*, the record pairs’ *blacklist*, the *synonyms*, and the *decision tree* of the T-match function.

**Figure 4:**
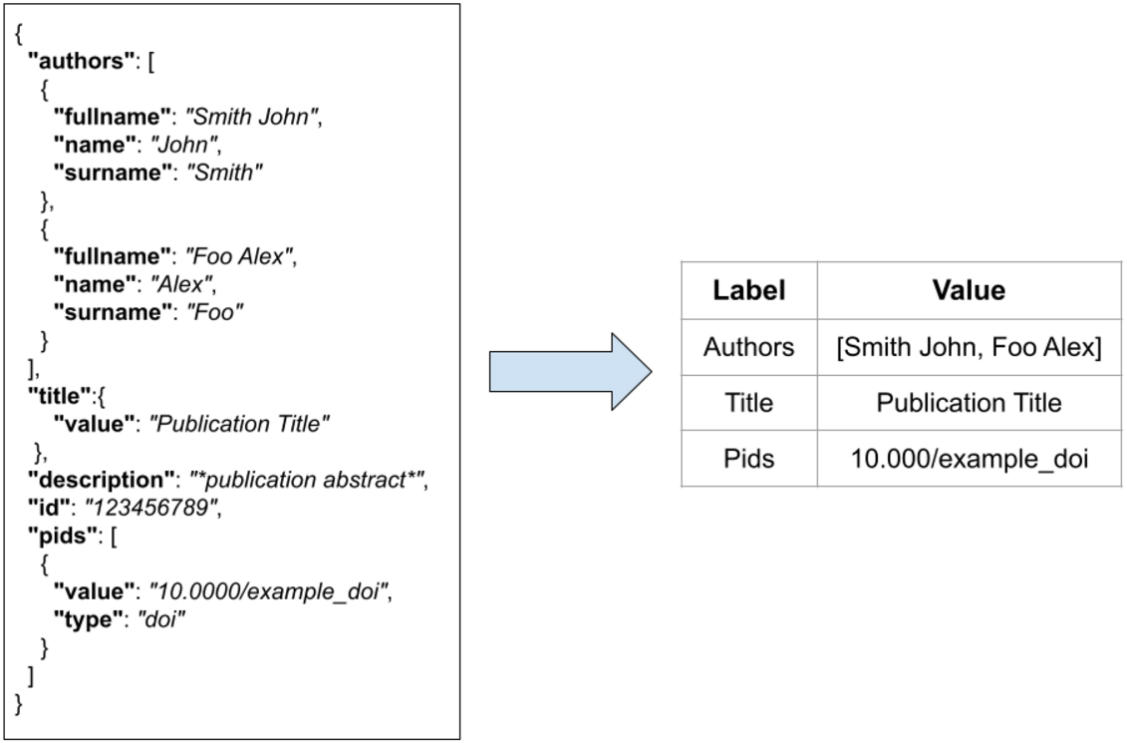
The transformation of an original JSON record into a flat record.

FDup operates on a collection of flat records with the same structure. As the original collection of JSON records may not be flat, FDup introduces the mechanism of the data model. The *data model* subsection of the configuration drives the transformation of the original JSON records onto a flat record with labels and values as depicted in Fig. 4. The mapping is defined by means of JSON paths, whose result is implicitly assigned to a given field of the FDup’s record data model. Additional parameters such as length and size can be used to limit the value to take from the original JSON entity.

The *clustering* subsection includes the list of blocking functions (more than one can be used) to be used for the key extraction. Each clustering function specifies the list of record fields to which the function should be applied. The user can also specify parameters for the clustering function such as key length, maximum number of keys to extract and other configurations. The structure of a clustering function is depicted in Table 1.

The *blacklist* is a list of field values to be excluded from the computation. The mechanism is useful to exclude false positives from subsequent rounds of deduplication. It is expressed as a map in which the key is the field name while the value is the list of strings to exclude. When the process is running, entities with one of those values for the specified field will be ruled out of the comparisons.

The *synonyms* are lists of equivalent terms. They are typically used to encode semantic equivalence across different vocabularies (*e.g.*, replacing terms with a common code). For example, synonyms are exploited to address translations of terms across different languages and therefore capture their equivalence.

The *decisionTree* section sets the configuration of T-match’s pair-wise comparison algorithm. T-match’s tree can be shaped up by creating different nodes, each composed by one or more comparators. Each comparator acts on a pair of records and over a given pair of fields, to return a number that reflects the similarity of two fields. The results of the comparators in one node are then aggregated to return an overall similarity score. The user can define the aggregation function to be used (*i.e.*, weighted mean, average, maximum, minimum, *etc*.). The structure of a comparator and a tree node are depicted in Table 2 and Table 3.

**Table 1:** Definition of a clustering function.

**Field**	**Description**
Name	The name of the clustering function
Fields	The list of fields to which the clustering function should be applied
Params	The list of parameters to configure the clustering function. Every parameter has a name and can assume a number as value. Those parameters are accessible from the clustering function.

**Table 2:** Definition of the comparator.

**Field**	**Description**
Field	The field to compare (it must be defined in the model)
Comparator	The comparator to use for the comparison
Weight	The weight of the score for the comparator. The value is used when the comparator’s scores are aggregated to give a weight to the comparator.
countIfUndefined	Boolean value that specifies if the score should be considered in the aggregation also when the result of the comparison is undefined (*i.e.*, missing field)
Params	The list of parameters for the comparator. Every parameter has a name and can assume a string as value. Those parameters are accessible from the comparator.

**Table 3:** Definition of the tree node.

**Field**	**Description**
Fields	The list of comparators contributing to the computation of the final score
Threshold	The threshold for the final score of the node. If the final score is greater than the threshold, the execution will follow the positive edge; otherwise, it will follow the negative edge. The execution will follow the undefined edge when the ignoreUndefined is not enabled and one of the comparators returned an undefined result.
Aggregation	The aggregation function to use for the computation of the final score (*e.g.*, maximum, minimum, average, weighted mean, ecc.)
Positive	The name of the next node to compute if the final score of this node is greater than or equal to the threshold.
Negative	The name of the next node to compute if the final score of this node is lower than the threshold.
Undefined	The name of the next node to compute if the result of the node is undefined.
ignoreUndefined	Boolean value that specifies if the undefined tree edge should be ignored or not.

##### Workflow section.

The section specifies parameters for the whole deduplication workflow. Such parameters are: *groupMaxSize*, *i.e.*, the limit for the block size, *slidingWindowSize*, *i.e.*, the size of the sliding window, *orderField*, *i.e.*, the field to be used when sorting the sliding window in a block, and *maxIterations*, *i.e.*, the maximum number of iterations when the connected components are computed via the close mesh operation.

#### Pace_Core module: Block Processor and T-match Processor

The BlockProcessor is the Java class that provides functionalities to process a block (*e.g.*, entity sorting), it implements the sliding window mechanism and it invokes the T-match Processor to performing the record pair-wise comparisons. The core of this class is the function that implements the queue management when blocks of records are sorted. Records in a block are queued in alphabetical order (of the *orderField*) and subsequently the queue is processed and pair-wise comparisons are computed.

The T-match Processor is the Java class that navigates the decision tree by calculating the result for every node according to the configuration provided in the Configuration file. Such class is invoked by the BlockProcessor and basically invokes each comparator involved in the node in order to collect and aggregate their scores (as specified by the user). At the end of the aggregation, a threshold is applied to the final score and the next tree node to process is chosen.

For the example in Fig. 2, when the T-match Processor is computing the score of the second node of the tree, it firstly calculates the scores for the two comparators (*i.e.*, *titleVersionMatch* and *sizeMatch*) and then aggregates them with an AND operation (*i.e.*, both conditions must be satisfied).

#### Pace_core module: libraries

As stated before, FDup offers a set of predefined and extendable libraries of comparators and clustering functions. They are implemented as Java classes inside the framework package.

The user can customize the framework by implementing new components depending on its needs. In order to implement a new comparator, it is sufficient to implement a Java class that extends the proper interface defined in the package.

##### Comparators.

In the context of the pair-wise comparison, the comparator specifies the logic that computes the score measuring the similarity degree of two fields of the pair, one for each record. Such a score is then aggregated with others in the same tree node to compute the overall similarity score of the node.

Comparators are Java classes that implement the comparison between two fields a and b of a record, whose Java interface is:

The only method to be implemented within the interface is compare. The method has three parameters: the two fields to be compared and the Configuration file, which drives the deduplication and may include comparators parameters. compare yields a double value in the range 0, …, 1 (0 different, 1 identical) indicating the similarity between the two field values. In particular, it returns 0 when the comparator cannot produce a result (*e.g.*, because one of the two labels is empty or missing) and a value in the range 0, …, 1 otherwise.

Table 4 describes the set of pre-defined comparators available in FDup today, which can be extended to address new application needs. In general, such a comparator framework grounds on the assumption that values are available via record fields. Hence, specific comparators can for example accept as input fields whose values are the result of smart pre-processing of the record collection, *e.g.*, machine learning embeddings, full-text extraction, topic modeling, *etc*.

**Table 4:** List of FDup comparators.

**Name**	**Description**
*AuthorsMatch*	Performs a “smart” comparison between two lists of authors; author names are matched by considering a custom similarity threshold and the result is the percentage of common elements between the two lists
*CityMatch*	Extracts city names from the field and returns the percentage of names in common; city names are matched by considering translations in different languages of the most important cities in the world
*ContainsMatch*	Searches for a given string in the input fields; logic operators (*i.e.*, AND, OR, XOR) can be used
*ExactMatch*	Returns 1 if the two fields are exactly the same. There are also other implementation of exact matches specific for a particular scope (*i.e.*, *DoiExactMatch* specific for DOIs, *DomainExactMatch* specific for URLs, and *ExactMatchIgnoreCase* to perform a case insensitive comparison)
*JaroWinkler*	Computes the JaroWinkler similarity function between two fields; the comparator can be specialized to address special cases, *e.g.*, *JaroWinklerNormalizedName* to compare two fields after removing city names and keywords, *JaroWinklerTitle* to compare fields containing titles, *SortedJaroWinkler* to a sorted version of the algorithm), *Level2JaroWinkler*, *Level2JaroWinklerTitle* and *SortedLevel2JaroWinkler*
*JsonListMatch*	Returns the common element percentage between two JSON lists extracted from the input fields
*KeywordMatch*	Extracts keywords from string fields and returns the common element percentage; the keywords are compared by considering translations in different languages provided via an input CSV; the list of keywords is customizable and located in the classpath
*Levenshtein*	Computes the Levenshtein distance measures between the two strings in the fields; possible specializations are *LevenshteinTitle* to compare specific title fields, *LevenshteinTitleIgnoreVersion* to compare titles and removing versions, and *SubStringLevenshtein* to compute the distance on substrings of the field, and *Level2Levenshtein*
*MustBeDifferent*	Returns 1 if the two fields are different
*NumbersMatch*	Extracts numbers from the input fields and returns 1 if they are equal
*RomansMatch*	Extracts Roman numbers from the input fields and returns 1 if they are equal
*SizeMatch*	Specific for lists, returns 1 if the size of two lists is equal
*StringListMatch*	Returns the percentage of common elements in two lists of strings
*TitleVersionMatch*	Specific for title fields, performs a normalization and returns 1 if numbers in the title are equal
*UrlMatcher*	Specific for URLs, performs a normalization of the URLs and returns 1 if they are equal
*YearMatch*	Extracts the year from the input fields and returns 1 if they are equal

##### Clustering functions.

Clustering functions are Java classes that implement the key extraction from a flat record. In the context of blocking, the clustering function specifies the logic that extracts the keys from the value of a certain label, *e.g.*, by computing ngrams, extracting the domain from an URL, *etc*. Such keys are subsequently used to group similar records into the same cluster and therefore limit the number of pair-wise comparisons.

In order to create a new clustering function, the following Java interface must be implemented:

The interface provides two methods:

• getParams: to access the list of parameters of the clustering function indicated in the configuration;
• apply: to produce the list of string keys extracted from the labels (*e.g.*, ngrams). The config parameter gives the user the possibility to implement comparators with access to the Configuration file.

FDup offers a list of pre-defined clustering functions, listed in Table 5. Such a list can be extended to introduce new approaches and strategies.

**Table 5:** List of FDup clustering functions.

**Name**	**Description**
*Acronyms*	Creates a number of acronyms out of the words in the input field
*KeywordsClustering*	Creates keys by extracting keywords, out of a customizable list provided in the classpath, from the field’s value
*LowercaseClustering*	Creates keys by lowercasing the field’s value
*Ngrams*	Creates ngrams from the field’s value; the number of ngrams and the length is indicated via parameters
*PersonClustering*	Specific for Person names, uses name and surname to create keys
*PersonHash*	Creates an hash of the Person name
*RandomClusteringFunction*	Creates random keys from the field’s value
*SortedNgramPairs*	Creates ngrams from the field’s value and then combines them in pairs
*SpaceTrimmingFieldValue*	Creates keys by trimming spaces in the field’s value
*SuffixPrefix*	Creates keys by concatenating suffixes and prefixes from words in the field’s value
*UrlClustering*	Creates keys for an URL field by extracting the domain
*WordsStatsSuffixPrefixChain*	Creates keys containing concatenated statistics of the field, *i.e.*, number of words, number of letters and a chain of suffixes and prefixes of the words

#### The Dedup_Workflow

This module implements the workflow depicted in Fig. 1 by building on the Apache Spark Framework, which allows to define and configure applications with high performance for both batch and streaming data. The framework can be used with different programming languages (Java and Scala in FDup) and offers over 80 high-level operators facilitating the realization of parallel applications.

The deduplication workflow is implemented as an Oozie workflow that incapsulates jobs executing the three steps depicted in Fig. 3, to compute: (i) the similarity relations (*SparkCreateSimRels*), (ii) the merge relations (*SparkCreateMergeRels*), and (iii) the groups of duplicates and the related representative objects (*SparkCreateDedupEntity*). More specifically:

• *SparkCreateSimRels*: uses classes in the Pace_Core module to divide entities into blocks (clusters) and subsequently computes *simRels* according to the Configuration file settings for T-match;
• *SparkCreateMergeRels*: uses GraphX library to process the *simRels* and close meshes they form; for each connected component, a master record ID is chosen and *mergeRels* relationships are drawn between the master record and the connected records;
• *SparkCreateDedupEntity*: uses *mergeRels* to group connected records and create the representative objects.

## Methods

This section describes the methods utilized to assess the performance of FDup when T-match can implement exit strategies, compared to a traditional approach where this technique is not exploited. The evaluation has been carried out using the metadata record collections used to populate the OpenAIRE Research Graph, where FDup is used as the core deduplication component.

The aim of the evaluation is to assess performance, not the precision of the deduplication results, which instead depends on the clustering functions, window functions, comparators, node thresholds, and ultimately on the context of use and quality of metadata.

The following sections introduce the OpenAIRE Research Graph, the specific set of metadata records used for the assessment, and describe the behaviour of T-match in two setting modes, by adopting exit strategies and by adopting a traditional approach, where all fields are matched before taking a decision.

### OpenAIRE research graph

**Figure 5:**
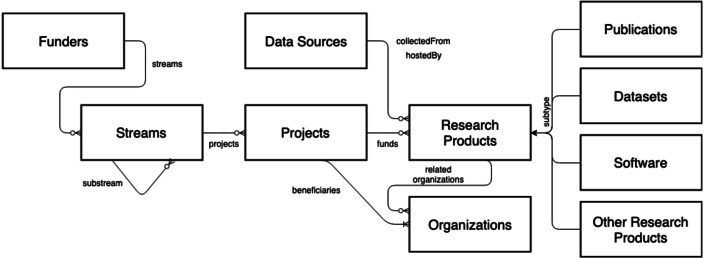
OpenAIRE Research Graph: data model.

The OpenAIRE AMKE is a no-profit legal entity (Manghi et al., 2012) whose purpose is to facilitate, foster, and support Open Science in Europe. The infrastructure has been operational for almost a decade and is successful in linking people, ideas, and resources for the free flow, access, sharing, and re-use of research outcomes. On the one hand, OpenAIRE manages and enables an open and participatory network of people willing to identify the commons and forums required to foster and implement Open Science policies and practices in Europe and globally. On the other hand, it operates a pool of technical services (OpenAIRE Catalogue, http://catalogue.openaire.eu) required to facilitate and monitor Open Science publishing trends and research impact across geographic and discipline boundaries. One of the core services of the infrastructure is the *OpenAIRE Research Graph*, a knowledge graph populated by harvesting, from 97,000+ data sources (*e.g.*, institutional, thematic, data repositories, ORCID, ROR, DataCite, Crossref, Unpaywall, MAG, *etc*.) and scientific journals, close to 300Mi+ metadata records and 1Bi semantic relationships among research entities, such as publications, datasets, software, organizations, projects, funders, authors, and data sources (for provenance); as a result of the deduplication process, the metadata records are merged into 150Mi+ representative records—for more details, visit http://graph.openaire.eu and http://explore.openaire.eu. A high-level view of the graph’s data model is depicted in Fig. 5.

FDup is today used as core component of the deduplication phase of the Graph, disambiguating metadata records of publications, datasets, software, other products, and organizations. The experiments carried out in this work rely on the subset of non-deduplicated publication records, for a total of 230Mi. The expriments will be performed over a sub-collection of 10Mi records and on the overall set of 230Mi. The collections are sufficiently large to appreciate the performance optimization gain introduced by T-match and ensure the replicability and reproducibility of the experiment.

### Experiment setting

The aim of the experiment is to show the performance gain yielded by the proper configuration of T-match in a deduplication workflow for the publication similarity match example presented in Fig. 2. To this aim, the experiment sets two deduplication workflows with identical blocking and sliding window setting but distinct T-match configurations. Both configurations address the similarity criteria but in opposite ways:

• ***PublicationTreeMatch* configuration**: a configuration that implements the *PublicationTreeMatch* decision tree illustrated in Fig. 2, taking advantage of early exits;
• ***PublicationWeightedMatch* configuration**: a configuration that implements the similarity match as the GDup (average mean) function *PublicationWeightedMatch* described in ‘Architecture’ by combining all comparators in one node, whose final result is a *MATCH* or *NO_MATCH* decision.

Both configurations are based on the same settings for the candidate identification and duplicates identification. In particular:

• the clustering functions used to extract keys from publication records are the *LowercaseClustering* on the DOI (*e.g.*, a record produces a key equal to the lowercase DOI, the result is a set of clusters composed by publications with the same DOI) and the *SuffixPrefix* on the publication title (*e.g.*, a record entitled *“Framework for general-purpose deduplication”* produces the key *“orkgen”*, the result is a set of clusters composed by publications with potentially equivalent titles); both functions are described in Table 5
• the *groupMaxSize* is set to 200 (empirically) to avoid the creation of big clusters requiring long execution time;
• the *slidingWindowSize* to limit the number of comparisons inside a block is set to 100 (empirically).

### Experiment methodology

Both the *PublicationTreeMatch* and the *PublicationWeightedMatch* configurations were performed over the publication record collection published in De Bonis (2021) (https://doi.org/10.5281/zenodo.5347803). The collection contains a set of 10Mi publications represented in JSON records extracted from the OpenAIRE Graph Dump (Manghi et al., 2021) (https://doi.org/10.5281/zenodo.4707307). In particular, publications have been selected from the Dump to form a dataset with a real-case duplication ratio of around 30% and a size that is appropriate to prove the substantial improvement in performance yielded by the early exit approach.

Two tests were performed, comparing the performance of the configurations *PublicationTreeMatch* and *PublicationWeightedMatch* over the 10Mi and the 230Mi collection respectively. The tests are intended to measure the added value of T-match in terms of performance gain, *i.e.*, *PublicationTreeMatch* vs *PublicationWeightedMatch* execution times.

The tests were performed with a driver memory set to 4 Gb, the number of executors to 32, the executor cores to 4, and the executor memory to 12 Gb. The Spark dynamic allocation has been disabled in order to ensure a fixed amount of executors in the Spark environment, so as to avoid aleatory behaviour. Moreover, since Spark’s parallelization shows different execution times, depending on both the distribution of the records in the executors and the cutting operations on the blocking phase, each test has been executed 10 times and the average time has been calculated.

The execution time was measured in terms of processing time required by *SparkCreateSimRels*, where the pair-wise comparisons are performed, and by *SparkCreateMergeRels*, where groups of duplicates are generated. It was observed that *SparkCreateSimRels* is dominant taking 70% of the overall processing time. As a consequence, for the sake of experiment evaluation, we: (i) reported and confronted the time consumed by *SparkCreateSimRels* under different tests to showcase the performance gain of T-match, and (ii) reported the results of the *SparkCreateMergeRels* to ensure that the tests are sound, *i.e.*, yield the same number of groups.

## Results

**Figure 6:**
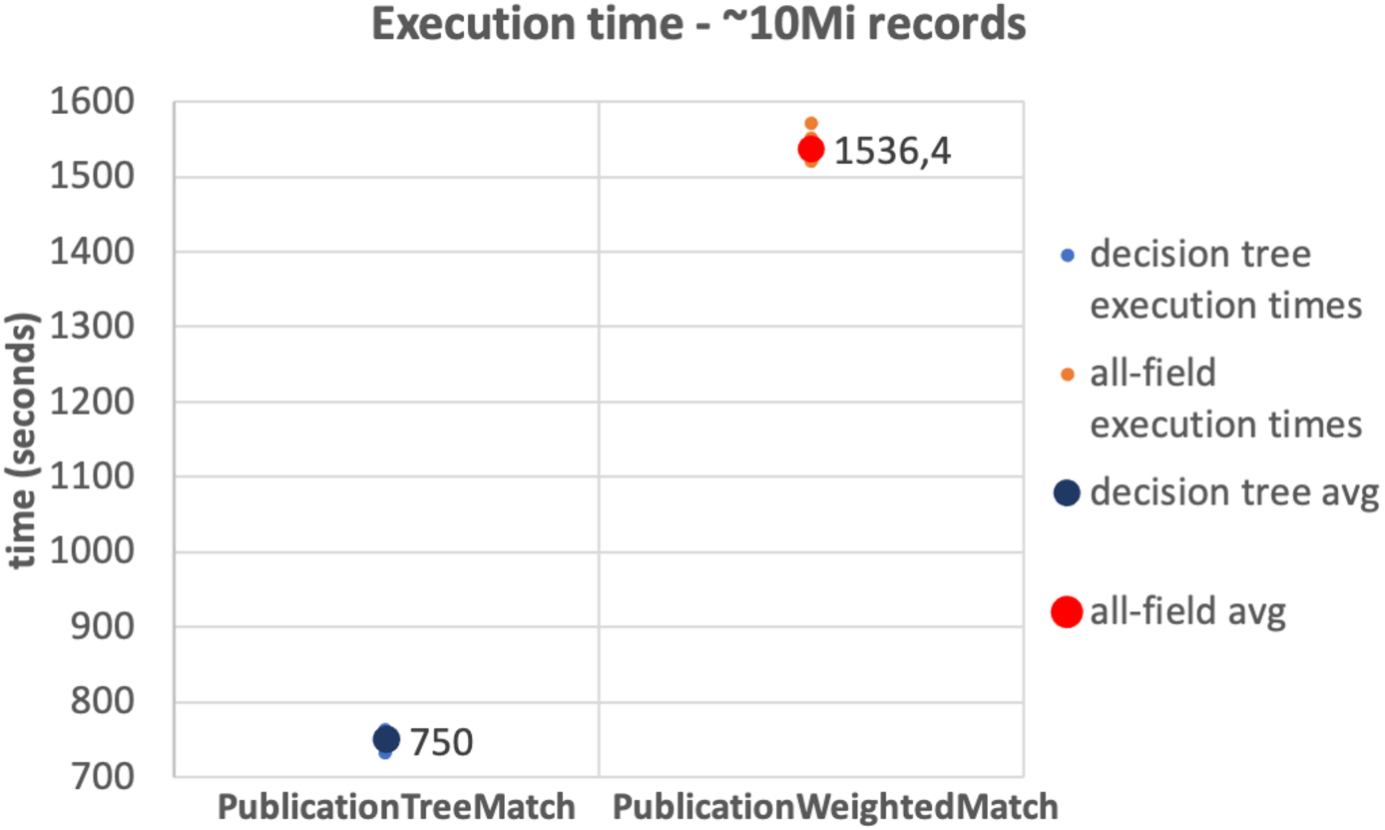
10Mi records test.

**Figure 7:**
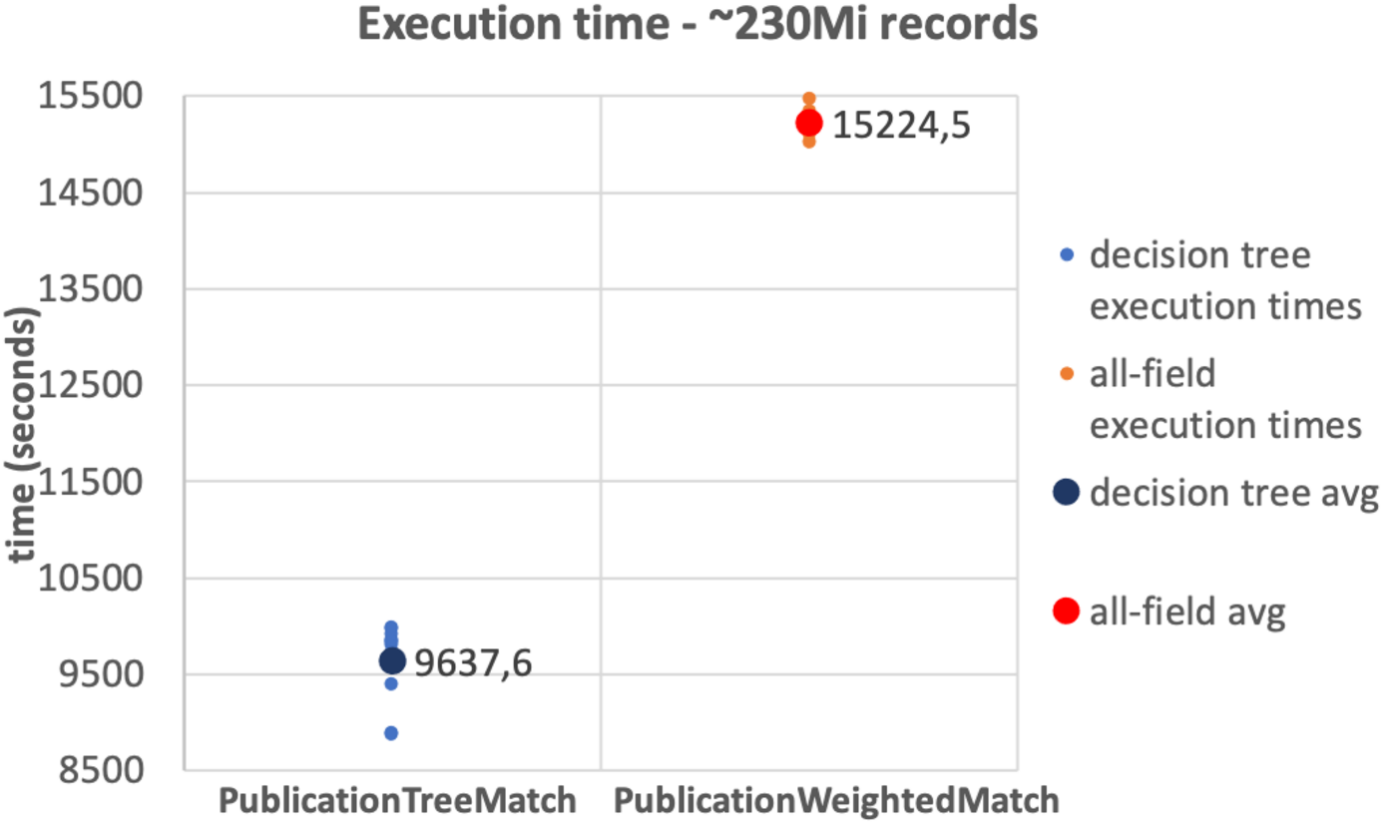
230Mi records test.

The results of the tests on the 10Mi publication records dataset and the 230Mi full publication datasets are depicted in Figs. 6 and 7, respectively. The graphs show the average time consumption of the *SparkCreateSimRels* phase for each execution of the test.

The average time of the *SparkCreateSimRels* stage in the test performed over 10Mi records dataset with the *PublicationTreeMatch* configuration is 750 seconds, while the *PublicationWeightedMatch* configuration consumes 1,536.4 seconds. The *SparkCreateSimRels* test on the 230Mi records dataset features an average time of 9,637.6 seconds for *PublicationTreeMatch* and of 15,224.5 seconds for *PublicationWeightedMatch*.

The results reported in Table 6 show that the two scenarios produced a comparable but not identical amount of *simRels*, *mergeRels* and *connectedComponent*. Differences are due to two main aspects: the size of the datasets, which required us to impose a limit to the block size to avoid uncontrolled execution time, and the Apache Spark behavior, which introduces a non-deterministic degree in the way blocks are formed (*i.e.*, keys are randomly distributed in parallel across blocks). These factors may introduce slight differences between the blocks resulting from different runs over the same input set. However, for both input datasets, the differences of *simRels* and *mergeRels* across different runs are limited to a range of 1,000–2,000 and are therefore not influential to the validation of the experiment. The differences between the two configurations is measured using the relative change, *e.g.*, the variation between the number of relations in terms of percentage.

**Table 6:** Average number of relations drawn by the deduplication workflow on 10Mi and 230Mi publication records.

**Size**	**Relation type**	**TreeMatch**	**WeightedMatch**	**Relative change (%)**
10Mi	*simRels*	13,865,552	13,866,320	0.000055
	*mergeRels*	5,247,252	5,247,585	0.000063
	*connectedComponents*	1,890,012	1,890,148	0.000071
	*pairwiseComparisons*	255,772,628	255,772,628	0.0
230Mi	*simRels*	172,510,072	172,511,772	0.0000098
	*mergeRels*	69,974,139	69,974,155	0.00000022
	*connectedComponents*	25,250,036	25,250,143	0.0000042
	*pairwiseComparisons*	3,650,733,202	3,650,733,202	0.0

Based on such results, it can be stated that the *PublicationTreeMatch* configuration overtakes the *PublicationWeightedMatch* configuration in terms of time consumption, by improving performance up to a 50% in the first test and up to 37% in the second test. The tests show a significant performance improvement, which suggests that the performance gain does not depend on the size of the dataset but improves with the number of early exits. It is also important to mention that the time measured in our tests includes the clustering phase. This may suggest that a notable amount of time is consumed by the key generation process especially when the input dataset is larger.

## Discussion

This work presented FDup, a framework for the deduplication of record collections that allows to: (i) easily and flexibly configure the deduplication workflow depicted in Fig. 1 and (ii) add to the known execution time optimization techniques of clustering/blocking and sliding window, a new phase of similarity match optimization.

### Flexibility and customization

**Figure 8:**
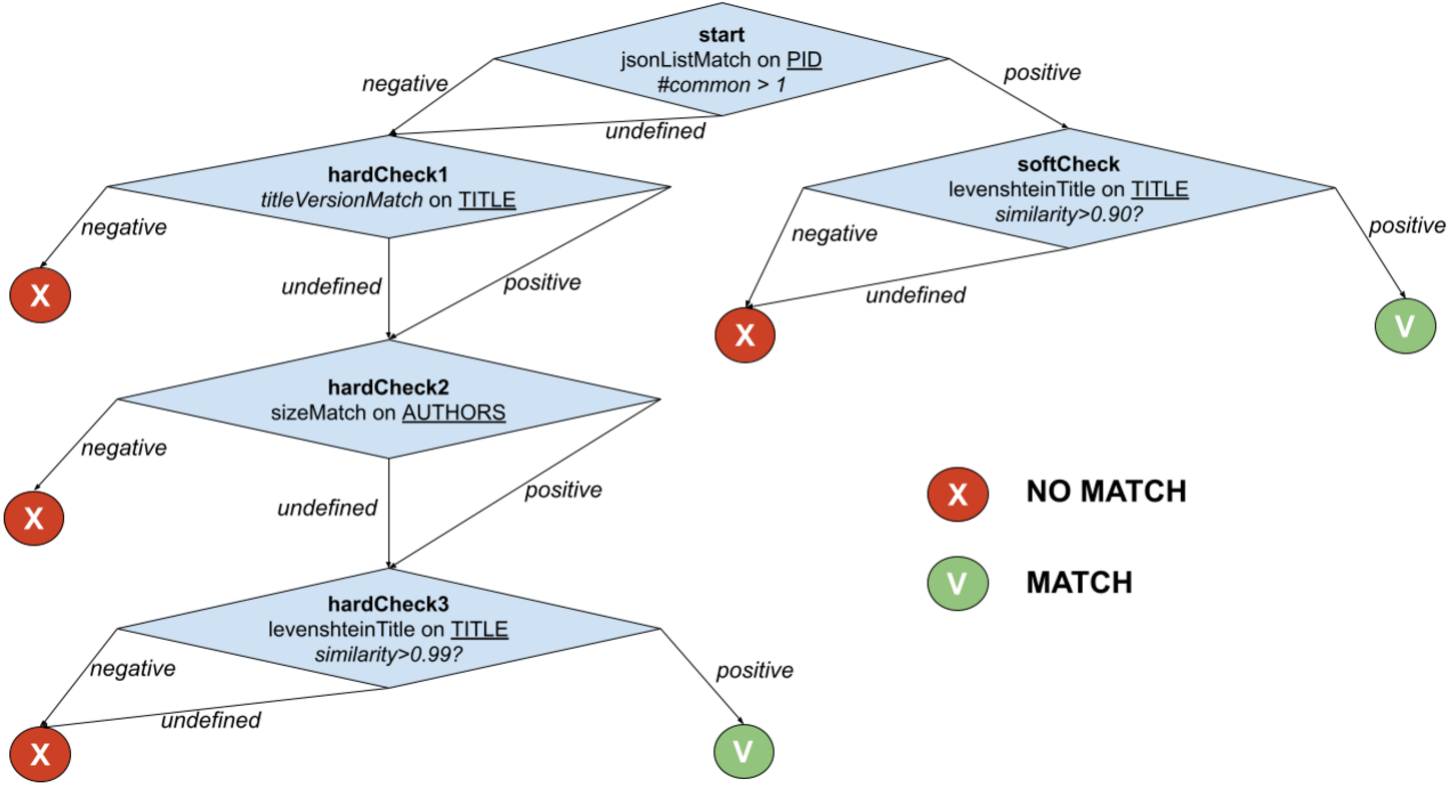
T-match’s decision tree for *DatasetTreeMatch*.

The framework allows to personalize a deduplication workflow by means of a configuration file and a rich set of available libraries for comparators and clustering functions. The record collection data model can be adapted to any specific context and the T-match function allows for the definition of smart and efficient similarity functions, which may combine multiple and complementary similarity strategies. For example, Fig. 8 shows a decision tree *DatasetTreeMatch* used to deduplicate research dataset records in the OpenAIRE Research Graph. The function mirrors the one used for publication records, but includes an extra path as the equivalence by identity requires stronger criteria in the case of datasets. In this case, the field PID may include values that are not related to the dataset but rather to the PID of the article that is related to the dataset (*e.g.*, *supplementedBy* relationship in DataCite’s ontology). Hence, in order not to merge datasets and articles, an extra test on the title is performed.

Encoding the functions of this kind by means of weighted means similarity functions is in general not possible. Furthermore, the readability and therefore reusability of a decision tree, with node names, edges, and *MATCH* and *NO_MATCH* nodes, are by far better than the ones of a mathematical function.

### Execution time optimization

The implementation of the entire FDup workflow by using Spark contributes to the optimization of the computation because of the parallelization of the tasks in the clustering and similarity checking phase. T-match gains further execution time by anticipating the execution of no-match decisions and postponing time-consuming decisions, such as the *AuthorsMatch* in the example. As proven by the reported experiments the hypothesis is not only intuitively correct but brings in some scenarios substantial performance gains. When used to analyse big data collections, time saving is key for many reasons: the execution of experiments to improve a configuration, speeding up the generation of quality data in production systems or saving time that can be spent to improve the recall and precision by relaxing clustering and sliding window approaches, *i.e.*, large numbers of blocks and increased window size.

On the other hand, time-saving depends on the nature of the input records and the ability to identify smart exit strategies applicable to a considerable percentage of the pair-wise comparisons. For example, if the publication record collection used for the experiments featured correct and corresponding PIDs for all records, the *PublicationTreeMatch*  execution time would be further improved; on the contrary, if no PIDs would be provided the execution time would increase and get closer to the one of *PublicationWeightedMatch*. The two functions would perform identically if, for all pair-wise comparisons, the records would always feature no difference in the versions and no difference in the title, making the *AuthorsMatch* title determinant to the final decision.

## Conclusions

FDup is currently in use in the OpenAIRE production system to deduplicate entities of various kinds, such as publications, datasets, software, organizations, and services. The deduplication criteria are often updated due to user feedback or natural refinements of the approach, proving the flexibility and usability of the framework. Still, a number of improvements are possible and currently under consideration to generalize the framework, to further optimize execution time, and to improve the quality of results.

*Generalization*: The framework is designed to perform comparisons between entities of the same record structure. It could be possible generalize the approach to enable comparisons between entities of different types, turning the framework into an entity linking tool by which not only relations for deduplication can be drawn but also semantic relations between records.

*Optimization*: Block purging and block filtering techniques can be integrated into the framework to further reduce the number of pair-wise comparisons within a block. Known approaches described in the survey from Papadakis et al. (2019) detect significant pairs based on the number of times the pair appears in multiple blocks. Edge filtering techniques can also be added to the framework to increase the precision of the deduplication. The general idea is to weigh similarity relations relying on the type of the match that determined their equivalence. For example, when a relation is drawn because of a match on a persistant identifier, its weight is higher than the weight of a relation drawn because of a match on the title.

*Quality of results*: Finally, new releases of FDup are exploring the possibility to post-process the resulting groups of duplicates to correct deduplication errors by computing statistics and by detecting the wrong element in a chain of similarity relationships, in order to split the affected group into two smaller groups. Such elements may be identified with algorithms based on deep learning and artificial intelligence and the group could be split by removing the less important relation that starts from the wrong element.

## Additional Information and Declarations

### Data Availability

The following information was supplied regarding data availability: The raw data is available at Github: https://github.com/miconis/fdup/tree/dnet-dedup-4.1.10. The dataset used for the experiments is available at Zenodo: https://zenodo.org/record/5347803#.YhdW7e7MJTY.
